# Quantitative Phase Imaging Techniques for the Study of Cell Pathophysiology: From Principles to Applications

**DOI:** 10.3390/s130404170

**Published:** 2013-03-28

**Authors:** KyeoReh Lee, Kyoohyun Kim, Jaehwang Jung, JiHan Heo, Sangyeon Cho, Sangyun Lee, Gyuyoung Chang, YoungJu Jo, Hyunjoo Park, YongKeun Park

**Affiliations:** 1 Department of Physics, Korea Advanced Institute of Science and Technology, Daejeon 305-701, Korea; E-Mails: kyeo@kaist.ac.kr (K.L.); kkh2580@kaist.ac.kr (K.K.); jeongjh513@kaist.ac.kr (J.J.); prism@kaist.ac.kr (S.L.); astralatom@kaist.ac.kr (Y.J.J.); skdurl20@kaist.ac.kr (H.P.); 2 Department of Bio and Brain Engineering, Korea Advanced Institute of Science and Technology, Daejeon 305-701, Korea; E-Mail: janihyo@kaist.ac.kr; 3 Department of Chemistry, Korea Advanced Institute of Science and Technology, Daejeon 305-701, Korea; E-Mail: kaisy08@kaist.ac.kr; 4 Hankuk Academy of Foreign Studies, Yongin 449-854, Korea; E-Mail: Peter0414@gmail.com

**Keywords:** microscopy, optical imaging, bio-photonics, quantitative phase imaging, cell physiology

## Abstract

A cellular-level study of the pathophysiology is crucial for understanding the mechanisms behind human diseases. Recent advances in quantitative phase imaging (QPI) techniques show promises for the cellular-level understanding of the pathophysiology of diseases. To provide important insight on how the QPI techniques potentially improve the study of cell pathophysiology, here we present the principles of QPI and highlight some of the recent applications of QPI ranging from cell homeostasis to infectious diseases and cancer.

## 1. Introduction

Cellular metabolism and activities play crucial roles in the pathophysiology of human diseases. Hence, the cellular-level understanding of the mechanisms of the diseases holds the key to unlocking the secrets of a number of diseases. Unfortunately, our understanding of cellular pathophysiology remains incomplete and has been stymied by limitations of traditional imaging techniques.

The past few decades witnessed the emergence of several novel optical imaging techniques developed in physics laboratories and their translation into the fields of biology and medicine. Among them, quantitative phase imaging (QPI) is one of the optical microscopic techniques which has been actively and widely investigated by many researchers, not only from scientific interest but also from its unique advantages over conventional techniques. Although more focus has been placed on technical development in the beginning, the fields of QPI have since grown to include various interesting biological studies, largely owing to the following capabilities:

- Quantitative imaging: optical phase delay information can be related to the physical and chemical properties of the sample quantitatively [1].
- Non-invasive and label-free imaging: no need to use dye or fluorescent proteins [1].
- Easy to be extended with other optical modalities [2, 3, 4 and 5].
- Numerical focusing by the propagation of a reconstructed field image [6,7].

This has opened the door for direct analysis of live cells and their pathophysiological alterations. Here we summarize the recent advances in QPI techniques focused on the study of cell pathophysiology. The research work, highlighted in this article, suggests that various QPI methodologies may play a crucial role in answering contemporary questions in the pathophysiology of cells and tissues which could, indeed, bring a substantial improvement in the understanding, assessment and treatment of diseases.

## 2. Principles of QPI

### 2.1. Two-Dimensional (2-D)QPI Techniques

**Figure 1.**
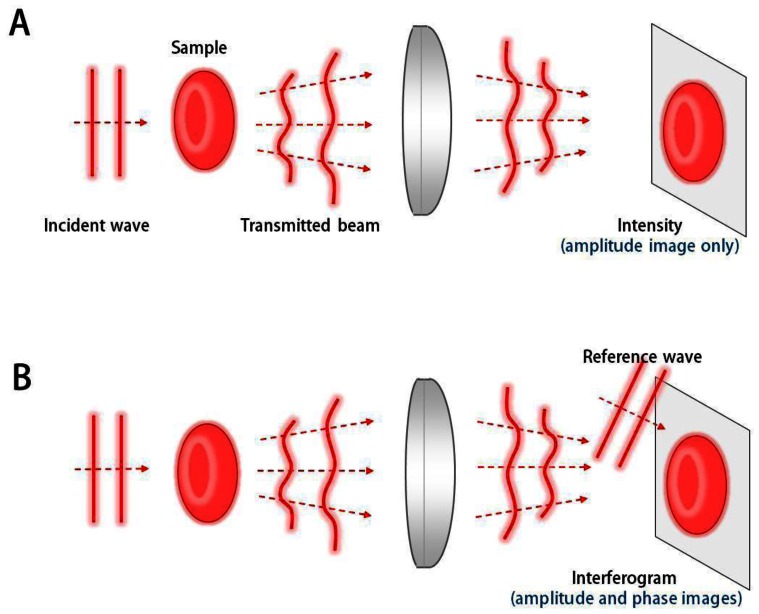
Principles of QPI (**A**) Conventional bright-field imaging measures amplitude information only; (**B**) QPI employs the principle of interferometry or holography, and measures both amplitude and phase information.

QPI techniques employ the principle of interferometry to measure the optical field, consisting of amplitude and phase information, whereas conventional bright-field imaging only measures the amplitude (Figure 1). Since most biological samples, including biomolecules, cells, and tissues, are optically transparent in visible light, information of amplitude does not provide good contrast for imaging. However, even these transparent samples provide significant optical phase delay, which serves as imaging contrast for QPI. Full details of the QPI techniques can be found elsewhere [1].

**Figure 2.**
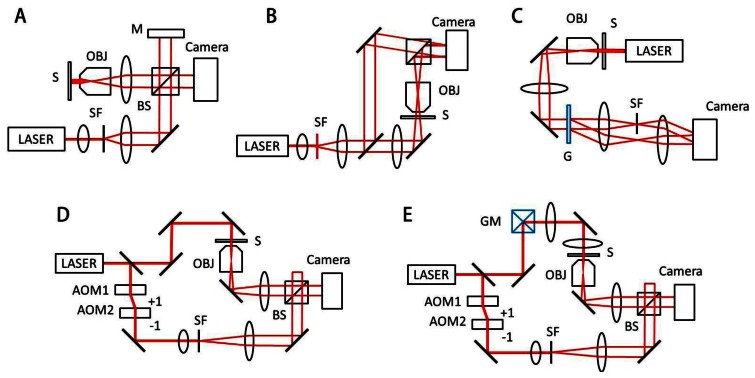
Experimental setups for typical QPI techniques. (**A**) Michelson interferometric microscopy; (**B**) Digital holographic microscopy, spatial-domain Mach-Zehnder interferometry; (**C**) Diffraction phase microscopy, spatial-domain common-path interferometry; (**D**) Time-domain Mach-Zehnder interferometric microscopy; (**E**) Tomographic phase microscopy, time-domain Mach-Zehnder type. The angle of illumination is controlled by a galvano-mirror (GM). AOM: acousto-optics modulator; SF: spatial filter; S: sample; OBJ: objective lens; BS: beam splitter; G; grating. (**A**–**E**) are modified from References [8, 9, 10 and 11], and [12], respectively, with permissions.

Generally, an interferogram or hologram, in which the optical field information of the sample is modulated with a reference beam (Figure 2), is digitally recorded by an imaging device such as a charge-coupled device (CCD), and the optical field information is then retrieved by appropriate field retrieval algorithms. Depending on the modulation method, QPI techniques can be mainly grouped into either the temporal modulation or spatial modulation.

Temporal modulation, or phase shifting interferometry (PSI), records sequential interferograms by changing the phase of a reference beam with respect to a sample beam. PSI can be achieved by translating a mirror in a reference arm with piezoelectric material or using acousto-optic modulation (Figure 2(D)) [13,14]. Since PSI requires the measurement of several interferograms in order to obtain one optical field image, the speed of QPI using PSI is limited by modulation speed.

QPI systems based on the Michelson or Mach-Zehnder interferometry (Figure 2(A,B)) have been widely used. QPI employing Michelson interferometer is suitable for the reflection geometry [15,16]. However, the Michelson or Mach-Zehnder interferometry suffers from time-varying phase noise due to vibration, temperature gradient, and air flow, which deteriorate the stability of QPI measurements. To minimize this phase noise, active noise control using feed-back loop [8] and common-path QPI utilizing spatial light modulator (SLM)such as the Fourier phase microscopy (FPM) [17] or spatial light interference microscopy (SLIM) [18] have been introduced. Quadriwave lateral shearing interferometry [19], which can be directly applied to conventional microscopy, also employs common-path geometry and demonstrates highly sensitive phase measurements.

In the spatial modulation scheme, a sample beam interferes with a reference beam and forms fringe patterns, from which the field information of the sample is retrieved. Depending on the way the fringe pattern is generated, spatial modulation can be divided into either the in-line holography or off-axis holography. In-line holography employs the interference of weakly scattered beam from a sample and an un-scattered incident beam [20] and optical instrumentation can be simplified with in-line holography. However, the optical field information is spatially overlapped with unwanted phase-conjugated information, or twin-image, and thus the field retrieval process for in-line holography involves computationally heavy iterative algorithms. In off-axis holography, the optical axis of a reference beam is slightly tilted with respect to the sample beam, which gives a well-defined carrier spatial frequency. Due to this carrier frequency, the twin-image information can be easily removed which provides a simple phase retrieval process [9]. In off-axis holography, only one hologram measurement is required to retrieve field information; the speed of QPI based on off-axis spatial modulation is mainly limited by camera speed [21].Digital holographic microscopy (DHM) is a typical off-axis holographic technique for the quantitative phase imaging of cells (Figure 2(B)) [6,9,22]. Hilbert phase microscopy (HPM) also employs off-axis holography and uses the Hilbert transformation for phase retrieval [21].

Diffraction phase microscopy (DPM) utilizes a diffraction grating to construct common-path interferometry with extremely high phase stability (Figure 2(C)) [10], which can be combined with fluorescence imaging channel [2]. The use of the SLM enables complex spatial modulation in QPI. In Laplace field microscopy [23] and gradient field microscopy [24], the scattered field at the Fourier plane is modulated by the SLM patterns of parabolic and sinusoidal shape, respectively. Similar to differential interference contrast (DIC) microscopy, they provide image contrast based on phase gradient of biological samples. However, the shadow artifact of DIC is eliminated in both Laplace and gradient field microscopy, and the enhanced edge profiles of samples are used for the study of dynamic membrane fluctuation. Spatial contrast microscopy employs a spatial phase plate at the Fourier plane and retrieved field information of biological samples [25].

Field retrieval algorithm is a practically important issue in QPI. In the temporal modulation scheme, once a set of holograms is obtained, a quantitative phase image can be directly extracted using phase-shifting algorithm [26]. In spatial modulation, Fourier transform [27] and Hilbert transform [21] have been used for phase extraction. Recently, spatial phase-shifting algorithm [28] and derivate method [29] have been developed to enhance the speed of field retrieval without using computationally intensive transformations.

Non-interferometric QPI can also be used which is based on the transport of intensity equation [30–34]. Non-interferometric QPI recorded a series of intensity images measured at different axial positions, from which a quantitative phase image is obtained with interactive phase retrieval process.

### 2.2. Three-Dimensional (3-D) QPI Techniques

A series of 3D QPI techniques have been developed to measure the 3D distributions of refractive index (RI) in biological cells, which offer a non-invasive means to probe the structural information of living cells without using exogenous labeling agents. They include interferometric methods to measure multiple transmitted optical fields with various angles of illumination (Figure 2(D)) [35–38], sample rotation [39,40], and wavelength scanning [41,42]. In addition, non-interferometric imaging technique [43] based on the transport-of-intensity equation [44] can also be used.

To reconstruct a 3D RI tomogram from multiple 2D images, an appropriate reconstruction algorithm is required. In the projection algorithm, analogous to X-ray computerized tomography, a 3D RI tomogram is calculated via the filtered back-projection method [12,39]. The projection algorithm works best for nearly transparent samples. However, diffraction algorithm should be used to take into account the diffraction of light induced by the sample. Diffraction algorithm, based on the first Born and Rytov approximation, assumes that the field scattered from the sample is only caused by the incident field. Technically, each 2D field map measured at different angles of illumination is mapped to the corresponding semicircular arc “Ewald surface” in the 3D Fourier space [36,45–47]. 3D optical tomograms obtained with the diffraction algorithm show high image quality with less distortion especially at defocused planes [48].

### 2.3. Extension of QPI to Other Areas of Investigation

QPI techniques can be extended with several optical modalities including spectroscopic and polarization-sensitive measurements. With Spectroscopic QPI, measuring field images at different wavelengths, molecular-specific phase information which is otherwise undetectable can be obtained *via* optical dispersion. Dispersion optical tomography uses two wavelengths and distinguishes gelatin solution from water [49,50]. Spectral-domain phase microscopy measures spectroscopic phase delay information employing optical coherence tomography where spectral information is readily available [51]. Spectroscopic phase microscopy combines color filters and a white-light source with DPM, which enables label-free quantification of hemoglobin (Hb) protein inside individual red blood cells (RBCs) [3]. A SLM can be used to select a specific wavelength [52]. Recently, it has been shown that one color hologram can provide three spectroscopic phase images, enabling dynamic spectroscopic QPI [53].

Polarization-sensitive QPI provides unique optical contrast for materials with birefringence such as chromosomes, spindle fibers, and collagen fibers. With two orthogonal polarized reference waves, birefringence was quantitatively imaged in a DHM setup [13]. The birefringence of a material is generally described using the Jones matrix; Jones phase microcopy measures the spatial distribution of Jones matrix of a transparent sample, recording four independent sets of polarization-sensitive quantitative phase images [54]. Recently, dynamic measurement of Jones matrix has been made possible by alternating the polarization state of incident beams and recording holograms simultaneously modulated with different orthogonal analyzer orientations [4].

QPI can also measure second harmonic generation (SHG) signals.SHG signals from the sample can be holographically recorded with a proper filter and a frequency doubler in a reference arm [55]. Some materials exhibiting intrinsic SHG signals such as collagen can be a primary target sample of SHG QPI [56]; other non-SHG materials can also be tagged with nanoparticles exhibiting SHG. In addition, when QPI is obtained in total internal reflection geometry (TIR), only small volume of the sample at the vicinity of the bottom surface can be imaged [57]. QPI can also be utilized for tracing spherical particles in 3-D space [58]. Furthermore, QPI techniques have also been successfully combined with the optical coherence tomography (OCT) [59,60], Raman spectroscopy [61], fluorescence [2,62], multi-photon excitation [63], and confocal microscopy [5]; these multimodal QPI techniques provide remarkable molecular specificity and thus provide wider window to investigate the biological processes.

### 2.4. Fourier Transform Light Scattering

With the optical field image measured by QPI, one can numerically calculate a far-field light scattering pattern of the sample by simply applying the 2-D Fourier transformation; this technique is called Fourier transform light scattering (FTLS) [64]. FTLS is the *spatial* equivalence of Fourier transform infrared spectroscopy (FTIR) that is related to the *temporal* frequencies. FTLS has unique advantages: (i) the scattering pattern over a broad angular range can be obtained in a single measurement; (ii) signal-to-noise ratio is extremely high due to the full utilization of image detectors; and (iii) light scattering pattern from *individual* micrometer-sized objects can be obtained.

Recently, FTLS has been used for the study of several biological studies [65,66]; light scattering from individual RBCs [67,68], microspheres [69], and colloidal clusters [70] have been investigated. FTLS was applied for the refractometry of spherical micro-objects in deep ultraviolet region [69]. Additionally, FTLS can be performedin conventional microscopy employing in-line holography without the relatively complicated holographic set-ups [71].

### 2.5. Light Sources for QPI

Most set-ups for QPI adopt coherent light sources to produce collimated beams and interference patterns easily. However, due to the long coherence length, QPI with coherent light sources suffers from unwanted speckle patterns (parasitic fringes), which deteriorate image quality and reduce phase sensitivity [1]. To overcome this issue, partially coherent light sources have been used. *Temporally* low-coherent light such as light-emitting device (LED) [6,72], Ti:sapphire pulsed laser [73], or even a white light source [74] can be used for QPI to reduce unwanted speckles. *Spatially* low-coherent light, which can be obtained by rotating a ground-glass [75] or illuminating a speckle field, can also significantly reduce speckle noise [76].

## 3. Study of Cell Physiology Using QPI

### 3.1. Structures of Cells and Tissues

**Figure 3.**
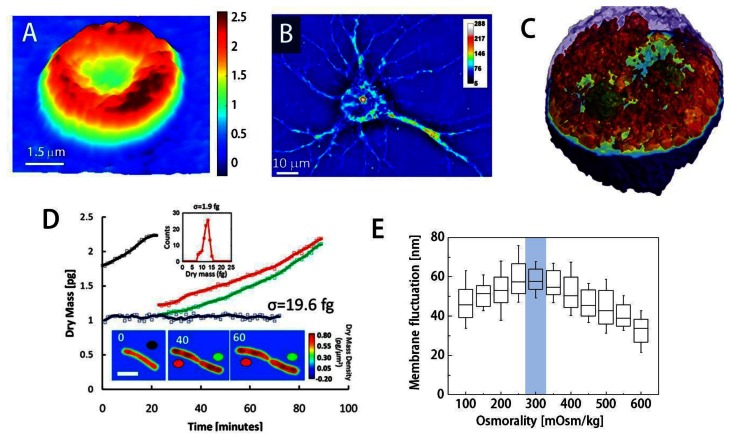
The study of cell physiology using QPI (**A**) Topography of a human red blood cell or erythrocyte measured by DPM; (**B**) A hippocampal neuron measured by SLIM; (**C**) 3D rendering of RI tomogram of a HT28 cell measured by TPM; (**D**) The growth of *E. coli* as a function of time measured by SLIM. Colored circles: growth curves for each cell; *inset*: single cell dry mass density at the indicated time points (in minutes) (Scale bar: 2 μm); *histogram*: the dry mass noise associated with the background (SD σ = 1.9 fg); blue line: a fixed cell; (**E**) Dynamic membrane fluctuations of human RBCs at various osmotic pressures. Blue area; physiological range of osmotic pressure. (**A**–**E**) are modified from Reference [79,18,12,82] and [83] respectively, with permissions.

Using QPI, researchers have demonstrated the label-free visualization and characterization of structures previously unobservable using conventional bright field microscopes. For instance, topography of individual red blood cells (RBCs) can be measured from phase images; the phase delay of RBCs can be directly translated into height information due to the lack of nucleus or sub-cellular organelles in RBCs (Figure 3(A)) [2,10,21]. Topography of RBCs is quantitatively and dynamically addressed without using labeling agents, which makes RBCs one of the most widely studied topics using QPI [77–80]. Besides the structural information, chemical properties of RBCs can also be obtained. The concentration of Hb can be simultaneously calculated by spectroscopic QPI [3,53], the combined analysis of the phase map and the bright field absorption measurements [81], and non-interferometric QPI [43].

QPI has also been applied to the imaging of neuron cells in culture: the height of neuron in culture [9] and the trans-membrane water fluxes [84] have been quantitatively studied using DHM. Cardiomyocytes have also been investigated using QPI *in vitro*: the spontaneous contraction of cardiomyocytes has been dynamically measured using wide-field interferometric microscopy [85]. The sensitivity of the analysis enabled the researchers to study the effects of temperature on the dynamics of the cellular contraction.

Despite advances, imaging general eukaryotic cells using QPI remained a challenge until the 3-D QPI techniques were developed since eukaryotic cells have complex morphologies with multiple subcellular organelles inside. Using QPI combined with confocal microscopy [5], Lue *et al.* have computed the integral RI of HeLa cells along the axial direction: the confocal microscopy measures the physical height distribution of the cell and it helped decouple the RI from the measured phase image. 3D RI tomograms of HT28 cells, *Caenorhabditis elegans*, and malaria-infected RBCs have been obtained with the tomographic phase microscopy (TPM) using the projection algorithm(Figure 3(C)) [12,78,86]. Recently, it has been shown that the TPM with the diffraction algorithm can provide high-resolution 3D RI tomograms; internal structures of HeLa cells [48] and the dry mass of chromosomes for colon cancer cell lines [87] have been reported. Spatial resolution of 3D QPI can be increased with deconvolution [88,89].

QPI has also been used to differentiate different types of *in situ* tissues by the average RIs [90], which can be used as diseases markers [91]. From 2-D field images measured with QPI, optical scattering parameters such as the scattering mean free path and the anisotropy factors can be calculated [92,93], which show promise in the optical diagnosis and prognosis of cancer tissues. Particularly, these optical parameters obtained with QPI have been used to identify sites of calcifications in breast biopsies, as well as to identify regions of malignancies in prostate biopsies [91].

### 3.2. Optical Measurement of the Dry Mass: Cell Growth and Division

Dry mass, the non-aqueous contents of cells which are mainly composed of protein, can be measured by QPI [94,95]. Optical phase delay is mathematically an integration of RI difference between the non-aqueous content of the cells through the cell thickness. Once the relationship between RI and dry mass contents (RI increment) is known, the phase image can be converted into dry mass of the cell. Because dry mass is independent of the water content inside the cell, it can be a good indicator for cell growth [95,82] and cell division [96] (Figure 3(D)).

Cell growth is a complex but highly controlled process; understanding the cell growth mechanism is crucial in cell biology with emphasis on cancer. Conventionally, mass of an individual cell is approximately estimated from its volume. Recently, techniques based on micro-channel or micro-electro-mechanical systems have been introduced [97], yet they remain technically complicated which prevents them from being widely used. The measurement of cell dry mass using QPI provides unique advantages for studying cell growth and division; cellular mass can be non-invasively and quantitatively measured with minimal perturbation; cellular mass can be monitored for a long period of time [95]. The dry mass of *E. coli* cells and human osteosarcoma U2O2 cells have been measured for more than 60 minutes, and the cycle-dependency of U2O2 cell growth has been reported [82]. Using the same method, the relationship between the motility and growth of S2 *Drosophila* cells under the influence of poly-L-lysine substrate has also been investigated [98].

### 3.3. Cell Dynamics

Dynamics of cellular activities and their alterations reveal the pathophysiological states of cells. For example, the RBC membrane cortex, composed of lipid bilayer, cytoskeleton and junctional protein complexes, exhibits dynamic fluctuations, also called “flickering.” Dynamic fluctuations in RBCs membrane had been an intriguing research topic in soft matter physics and hematology [99] after their first observation [100], and they have shown strong correlation with the pathophysiological states of the cell [99]. QPI is an ideal tool to probe the dynamic membrane fluctuations in RBCs since the phase images of RBCs can be directly and quantitatively translated into height map, measured at high speed [77,101,102]. Using QPI techniques, RBC membrane fluctuations have been investigated; fluctuation coherency [103], a sub-domain in the fluctuations [104], and its dependency of ATP has been analyzed [79,105]. A systematic study using QPI has also revealed that the dynamic membrane fluctuations in RBCs result from both thermal energy and metabolic energy variations stemming from the ATP phosphorylation process [79], which is also strongly related to the maintenance of the biconcave shape and the remarkable deformability RBC [79,106].

Biomechanical properties of RBCs can be calculated from the measured dynamic fluctuations in the cell membrane [107], and thus membrane tension [108] and effective viscoelasticity [109,110] have been studied. Recently, combined with a mathematical model, four important mechanical properties (bending modulus, shear modulus, area expansion modulus, and cytoplasmic viscosity) were simultaneously measured with QPI [80]. This method has been used to address the effects of osmotic pressure [83], malaria infection [78,86], and sickle cell disease [111,112].

Besides RBCs, dynamics of other cell types have also been investigated. For instance, QPI analyzed the voltage-dependent nanometer scale movements of nerve cells [113] and also measured the membrane motions of HEK 293 that is genetically modified to express prestin motor proteins [11]. Cell dynamics associated with electromotility have been further studied by QPI with a low-coherence light source [73]. Dynamic motion during the migrations of human dendritic cells [114] and subcellular contraction of embryonic cardiomyocyte [115] have also been studied using QPI.

One of the powerful advantages of QPI is that there is no need to use exogenous labels, and this allows easier sample preparation and more efficient measurement free of photo-toxicity and photo-bleaching. For example, the intracellular transport of ATP-consuming cargo along actin filament in the neuron cell has been studied using QPI data [116]; the spatio-temporal aspects of actin-driven dynamics in live glial cells have also been measured employing QPI and FTLS [117].

### 3.4. Homeostasis

Many researchers have used QPI techniques to investigate cell homeostasis. Using DPM, the effects of osmotic pressure on RBC morphology and deformability have been studied [83]. The membrane fluctuations of RBCs mark the maximal value at physiological osmolality, reflecting normal blood cells' high deformability when compared to those surrounded in hypotonic or hypertonic medium (Figure 3(E)). Furthermore, retrieved mechanical properties of RBC membrane also emphasize this point; shear and area compression modulus decrease until the osmolality reaches the physiological level (300 mOsm/kg) [83].

Cell morphology of the mouse cortical neurons, in which dysregulation of the intracellular calcium ion homeostasis was induced by excessive glutamate pulses, has also been investigated with DHM [84]. This reveals that the transient increase in calcium ion leads to a phase signal drop, which corresponds to cell swelling and decrease of the RI [62].

Constant gravitational field imposed on cells can also be regarded as a homeostasis condition. It is known that the simulated microgravity established by random positioning machine induces significant disorganization of cytoskeleton in less than an hour but these glial cells recover within a day. The real-time DHM setup enables us to dynamically observe these cytoskeletal modifications of mouse C2C12 myoblasts under the influence of microgravity [118].

### 3.5. Other Physiological Effects: Cell Death, Traction Force, Etc

QPI could be used to determine cell viability much faster than the conventional trypan blue staining test, which takes about several hours. Cell-death, either as apoptosis or necrosis, can be determined by measuring the mean phase shift using QPI. Mouse cortical neurons were induced apoptosis by applying L-glutamate, and the corresponding mean phase shift values for those cells have shown strong correlation with cell viability [119]. QPI can also be used to measure the traction force applied by fibroblasts during migration. The degree of wrinkling, introduced to the soft substrate by the contractile motion of the cell above it, has been quantitatively measured by QPI, from which the corresponding transition force was estimated [120].

Femtosecond laser photoporation, creating small holes in the cell membrane due to the radiation energy, utilizes the DHM [121]. The dynamics of CHO-K1 cells in response to femtosecond laser photoporation show that the radiation energy for photoporation correlates with the degree of temporal dynamics of the cell, which was observed from the change in the optical path length in the region of interest [121]. QPI has also been combined with laser microsurgery in order to evaluate the damage or repair of cells or organelles in real time during laser micro-dissection [122]. As another useful application of QPI, high speed cinematic DHM has been used to quantify the fast motility of dinoflagellates since the substantial depth of field of QPI allows simultaneous 3-D tracking of the swimming of many cells in dense medium [123]. Recently, using an in-line DHM, the Brownian diffusion of colloidal aggregation formed by short-range attraction has been studied [124], which suggests that the dynamics of complex biological system including cell aggregation or bacterial clusters might be systematically addressed using QPI.

## 4. Study of Cell Pathology

### 4.1. Infectious Disease

The same properties which made QPI such an attractive candidate for the study of cell physiology also enable the thorough study of human dieases. Especially, the label-free imaging capability of QPI makes it an effective optical imaging technique to quantitavely analyze the structure and dynamics of *Plasmodium falciparum* invaded-RBCs (*Pf*-RBCs) [125]. For over a century, optical imaging with Giemsa stain has been the “gold standard” for malaria diagnosis.Recently, however, it has been shown that RI information measured by 3D QPI can be used as an effective indicator to quantitavely analyze the physical and chemical alterations in *Pf*-RBCs.

**Figure 4.**
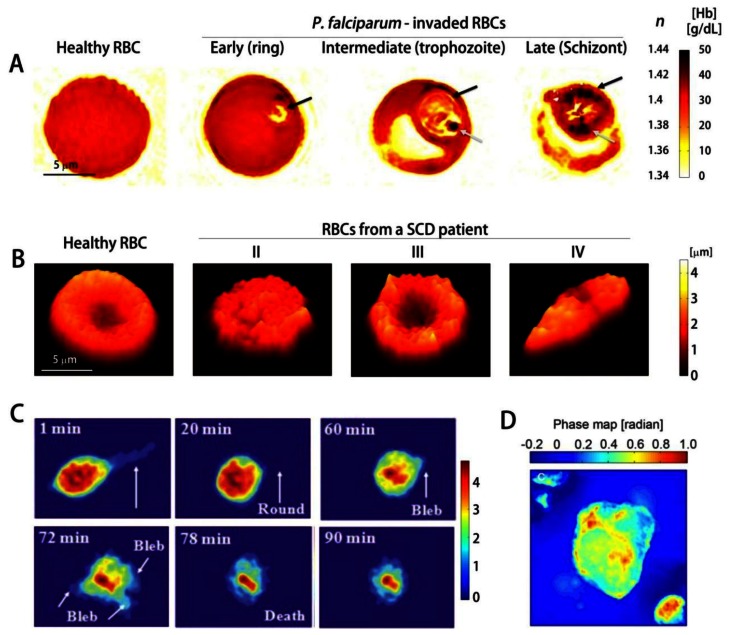
The study of cell pathology using QPI (**A**) Cross-sectional images of 3-D RI tomograms of *Pf*-RBCs measured by TPM. Black arrows: parasitophorous vacuole; gray arrows: hemozoin; (**B**) Topograms of RBCs from a SCD patient, measured by DPM. II-IV: classification based on morphology; (**C**) morphology of rat basophilic leukemia RBL-2H3 cells infected with *V. vulnificus* strains; (**D**) Phase image of high-definition-CTC measured by non-interferometric quantitative phase microscopy. (**A**–**D**) are modified from refs. [78,112,127], and [132], respectively, with permissions.

While healthy RBCs show uniform RI distribution, *Pf*-RBCs are optically inhomogeneous; they have vacuoles of parasites with low RI and the hemozoin crystals, insoluble polymerized forms of heme with high RI.These RI information can be used to determine the Hb concentration (Figure 4(A)) [78]. Using 3D QPI, the dynamics of the egress process of malaria infection have been investigated [86], which demonstrated that the inhibitors E64d and EGTA-AM prevent the merozoites from escaping host *Pf*-RBCs. Utilizing the DPM in combination with genetic knock-out technique, it has been shown that*Pf*155/Ring-Infected Erythrocyte Surface Antigen (RESA) are responsible for the decreased dynamic microcirculatory behavior of ring-stage *Pf*-RBCs [126]. In addition, analyzing the light scattering of the individual *Pf*-RBCs through the DPM and FTLS techniques, the specific disease state of *Pf*-RBC were identified and the alterations in mechanical properties of the cell membranes were determined [68].Other than malaria infection, QPI has been used for studying the morphology and volumes of rat basophilic leukemia RBL-2H3 cells infected with *V. vulnificus* strains (Figure 4(C)) [127], and the cellular damage induced by Shiga toxin in human brain microvascular endothelial cells, which results in haemolytic uraemic syndrome caused by enterohaemorrhagic *E. coli* [128]. QPI can also be used for the study of cytotoxicity assessment [129].

### 4.2. Genetic Disease: Sickle Cell Disease

QPI also proves to be highly useful in studying genetic diseases such as sickle cell disease (SCD). SCD is an inherited autosomal recessive genetic blood disorder, characterized by the abnormal deformation of RBCs especially under deoxygenation. This reduced deformability is mainly caused by polymerization of Hb inside RBCs and its implications. Characterizing mechanical properties at the single-cell level is a crucial step in comprehensive understanding of SCD [130]. Conventionally, several techniques including micropipette aspiration, optical tweezers, parallel-plate flow chamber method, and atomic force microscopy have been used. However, these methods have limitations: large probing force inevitably changes intrinsic mechanical properties of the cells and several important mechanical properties of the cells can not be retrieved simultaneously. Recently, QPI techniques have been employed to study the morphology and dynamic membrane fluctuation of the sickle RBCs [112,131]. Dynamic membrane fluctuations of sickle RBCs were significantly lower than healthy RBCs, indicating the reduced deformability. In addition, several key mechanical properties of sickle RBCs were able to be determined from the measured dynamic membrane fluctuation (Figure 4(B)) [112]. Static and dynamic light scattering signal from individual sickle RBCs have also been analyzed using DPM and FTLS techniques [111].

### 4.3. Cancer

QPI has also been used to study cancer cells, and has proven its capability to non-invasively study the effects of various chemicals to cancer cells, which could yield insights into the progression of cancer.Using a non-interferometric QPI system, the cellular dry mass of circulating tumor cells (CTC) and leukocytes have been characterized (Figure 4(D)) [132]; CTCs are more massive and heavier than leukocytes. In addition, QPI has also enabled characterization of the morphology and the average RI of pancreatic cancer cell lines PaTu9899 [133]. Furthermore it has also enabled the observation of the dynamic changes in the morphology upon the addition of the cell toxin Latrunculin B, which showed that cell heights decrease due to a breakdown of actin filaments. In addition, the quantitative phase images of *in-situ* tissue biopsy samples can also be used for identifying the regions for tumors and calcifications, since they contain light scattering information which reflects cellular and subcelluar structures [91].

## 5. Conclusion and Outlook

Here, we have summarized recent advances in QPI techniques which offer powerful advantages over the traditional optical imaging methods. The recent development of various QPI techniques has shown great potential for translation into the fields of cell biology, biophysics, analytical chemistry, and medicine. Yet, the usage of QPI techniques has not been fully explored. Many important challenges are still left and are now to be tackled with the advance and the clever usage of novel QPI techniques. For example, the implementation of QPI techniques into conventional optical microscope systems and enabling the easy usage by non-specialists are necessary. In addition, on-chip technique [134] can miniaturize the QPI methodologies, which potentially enables point-of-care diagnosis and treatment of various diseases in a portable and disposable platform. Moreover, the improvement in spatial resolution of QPI would enable the access to the structures and sub-cellular organelles of biological cells with the advantages of QPI. Recently, several techniques for high-resolution QPI have been demonstrated including speckle-field illumination [76], synthetic aperture microscopy [135], sparse deconvolution [89], and phase nanoscopy using a quasi-2π-holographic detection and deconvolution [136]. Furthermore, integrated with existing fluorescence-based super-resolution microscopic techniques [137,138], QPI can also be utilized to reveal the molecular-level alterations of cell disease states.

In the near future, QPI techniques would be integrated with wavefront shaping to study biological cells and tissues *in vivo*. Currently, most of the QPI techniques address the biological specimen *in vitro* or *ex vivo*. This is because multiple light scatterings arise when light passes through a biological tissue with inhomogeneous RI distribution. Recently, holographic wavefront shaping techniques have shown interesting results which can control and suppress multiple light scattering events so that optical imaging can be delivered though highly turbid media [139,140]. Since wavefront shaping in turbid media enables the manipulation of light in spatial [141], temporal [142], spectral [143], and polarization-dependent domains [144], the combination between QPI and the wavefront shaping techniques would be a powerful tool to investigate the structures and dynamics of cells. Furthermore, direct *in vivo* QPI image through turbid tissue layers [145] would provide a means to probe the states of cells in their most intact conditions.
